# Overdose effect of aconite containing Ayurvedic Medicine (‘*Mahashankha Vati*’)

**DOI:** 10.4103/0974-7788.72493

**Published:** 2010

**Authors:** Ashok Kumar Panda, Saroj Kumar Debnath

**Affiliations:** *Ayurveda Regional Research Institute, Tadong, Gangtok, Sikkim – 737 102, India*

**Keywords:** Bradycardia, hypotension, *Mahashankha Vati*, overdosing, *Vatsanabha* root

## Abstract

There are chances that the use of larger than recommended dose of Ayurvedic medicines containing aconite can produce drug reactions. *Vatsanabha (Aconitum ferox* Wall.) is a very well-known ingredient of Ayurvedic formulations and is prescribed as an antipyretic, analgesic, anti-rheumatic, appetizer and digestive. The recommended dose of purified *Vatsanabha (A. ferox* Wall.) root is 15 mg. We present a case of hypotension and bradycardia due to aconite poisoning caused by overdosing of an Ayurvedic medicine (*Mahashankha Vati*), which was primarily managed by Ayurvedic treatment.

## INTRODUCTION

It is a common misconception among the public that Ayurvedic medicines are safe and devoid of adverse reaction. More than 70% of the total sales of Ayurvedic drugs are over-the-counter (OTC), thus leading to the use of Ayurvedic medicines without prescription, guidance and oversight of Ayurvedic physicians.[1] Many poisonous plants like *Ahiphena (Papaver somniferoum* Linn.), *Bhanga (Cannabis sativa* Linn.), *Dhattur (Dhatura metel* Linn.), *Karavira (Nerium indicum* Mill.), *Kupilu (Strychnos nuxvomica* Linn.f.), *Langali (Gloriosa superba* Linn.), *Vatsanabha (Aconitum ferox* Wall.), *Jayapal (Croton tiglium* Linn.), etc. have been used in Ayurveda medicine.[2] According to Ayurveda, “even a strong poison can become an excellent medicine if administrated properly; on the other hand, even the most useful medicine can act like a poison if handled incorrectly”.[3] Unexpected adverse reactions can occur due to accidental use of a poisonous herb/medicine/decoction by the patient, misidentification of herbs so that a toxic herb is mistaken to be a harmless variety, improper purification of the poisonous ingredients, overdose, irrational prescription, self-medication and drug interaction with allopathic drugs.

Aconite-based Ayurveda medicines are commonly used by Ayurvedic physicians and traditional practitioners/folk healers in primary healthcare. The most common aconite-based medicinal plant *Vatsanabha (A. ferox* Wall.) is used in Ayurveda as an antipyretic, analgesic, anti-rheumatic, appetizer and digestive.

Aconite poisoning following use of herbal remedies has been reported from Hong Kong, India and Nepal.[4–6] Management of aconite overdosing is supportive, including immediate attention to the vital functions and close monitoring of blood pressure and cardiac rhythm. Inotropic therapy is required if hypotension persists and atropine should be used to treat bradycardia.[7]

We present here a case of hypotension and bradycardia due to overdose of aconite-based Ayurvedic medicine.

## CASE REPORT

A 47-year-old male reported to the OPD with the complaint of numbness around the mouth, nausea, heaviness of tongue, vertigo, black out, bursting type of pain over the back of the head, numbness and tingling sensation of palm and sole, and severe sweating which gradually reduced. The blood pressure was recorded as 30/10 mm Hg and pulse rate was 46 per minute with irregular rhythm, low volume and low tension. The extremities were cold and clammy, and there was retention of urine. There was no history of loss of consciousness or seizure. The patient was oriented and alert. The patient was a known case of hypertension but he had not taken antihypertensive drugs for the last 10 days. Five years ago, the patient had suffered from peptic ulcer syndrome.

The patient had taken two tablets of *Mahashankha Vati* along with two tablets of *Citrakadi Vati* without the advice of Ayurvedic physician or pharmacist of the institute, following complaints of abdominal pain and heaviness in the abdomen. A provisional diagnosis of aconite poisoning due to overdose of aconite containing Ayurvedic medicine (*Mahashankha Vati*) was made and the patient was admitted for observation.

Emesis was induced with salt water. Following this, 1 g of *Tankan bhasma* (sodium biborate) and 1 g of *Maricha Churna (Piper nigrum)* was given orally with 50 ml of water. After 30 minutes, one glass of lukewarm milk with ½ tablespoon of ghee and 1 tablespoon of honey was given, followed by 20 ml of *Arjunarista* and 20 ml of *Kanakasava* with 40 ml of water. Thirty minutes later, the blood pressure was 60/30 mm Hg and pulse rate was 60 per minute with regular rhythm, low volume and low tension. Three hours later, the blood pressure and pulse remained the same and patient complained of severe fatigue and lassitude. Two bottles of 5% dextrose with normal saline (DNS) and one bottle of 10D (10% dextrose) along with 8 mg dexamethasone were given intravenously. Two hours later, the blood pressure was 100/60 mmHg and pulse was 70 per minute with regular rhythm, low volume, low tension. The patient had passed urine.

At this time, 20 ml of *Arjunarista* and 20 ml of *Kanakasava* were given with 40 ml of water along with 1 g of *Tankan bhasma* (sodium biborate) and 1 g of *Maricha Churna (Piper nigrum)* with 50 ml of water. Before these medicines were given, the patient had a light dinner of semi-liquid rice (*Manda*) and salt. The patient was discharged the next day after full recovery. He was prescribed 20 ml *Dasamularista* and 20 ml *Punnavarista* twice daily to be taken after meal with 40 ml of water to assist in the renal clearance of the aconite and to bring the *tridosha* into equilibrium state. Two capsules of Arjun (each capsule containing 250 mg of crude extract of Arjun) twice daily in the morning and evening after intake of food for 3 days.

## DISCUSSION

*Vatsanabha (A. ferox* Wall.) is a very well-known ingredient of Ayurvedic formulations and is prescribed as an antipyretic, analgesic, appetizer and a digestive. However, *Vatsanabha* root is always used after purification in these preparations. The process of purification involves submerging the roots into the fresh urine of a healthy cow for 3–7 days. Then, the outer covering of the root is pealed off and cut into small pieces and left in the sunlight for 1 day. After this, it is boiled with fresh cow milk for a minimum of 3 hours. Finally, it is dried in sunlight and powdered. The recommended dose of purified *Vatsanabha (A. ferox* Wall.) root is 15 mg.^7^ It is used in *Tridosaja vikara*, especially in *Kapha vataj roga. Vatsanabha (A. ferox* Linn.) is indicated in skin diseases, inflammatory diseases, anorexia, cough, bronchitis, fever, pain, rheumatic condition, and as an aphrodisiac also. The very popular classical Ayurvedic formulations containing *Vatsanabha* are *Mrutunjaya rasa, Saubhgya vati, Rambana Rasa, Kaphaketu Rasa, Sanjeevani Vati, Moha Shankha Vati*, etc.[8] *Vatsanabha* has significant therapeutic action in clinical and experimental studies.[9,10] According to Thorat and Dahanukar, “Crude aconite is an extremely lethal substance”. However, the science of Ayurveda looks upon aconite as a therapeutic entity. Crude aconite is always processed, i.e., it undergoes “*samskaras*” before being utilized in the Ayurvedic formulations. This study was undertaken in mice to ascertain whether “processed” aconite is less toxic as compared to the crude or unprocessed one. It was seen that crude aconite was significantly toxic to mice (100% mortality at a dose of 2.6 mg/mouse), whereas the fully processed aconite was absolutely nontoxic (no mortality at a dose even 8 times as high as that of crude aconite), and further, all the steps in the processing of aconite root were essential for complete detoxification.[11] Pharmacological studies reveal purification of aconite after *Shodhana*.[12,13]

The tuber of *Vatsanabha* contains 0.4–0.8% diterpene alkaloids and the concentration of aconite in the fresh plant is between 0.3% and 2.0% in tubers and 0.2% and 1.2% in the leaves. The highest concentration of aconite is found in the winter.[14] The major alkaloids are aconitine, pseudaconitine, bikhaconitine, diacetyl pseudaconitine, aconine, picro-aconine, veratry pseudaconitine, chamaconitine, veratryl gama aconine, and di-Ac–Y-aconitine.[15] Aconitine and related compounds exhibit antipyretic, anti-inflammatory, analgesic and anti-rheumatic properties in experimental models and certain species of aconite have antitumor activities and regulate neurological disorders.[16]

Aconite is a strong poison affecting several systems. Pure aconite can cause death at a dose of 2 mg, while 1 g of the aconite plant is fatal. Aconite toxicity is characterized by a burning or tingling sensation of the lips, tongue, mouth, and throat almost immediately after ingestion. Numbness of the throat and difficulty with speech may ensue. Nausea, vomiting, dizziness, fatigue, blurring of vision and paresthesia may also occur. Toxicity mainly affects CNS, heart and muscle tissues, primarily resulting in cardiovascular complications. Several case reports describe poisoning with aconite or its constituents, resulting in ventricular tachycardia, ventricular fibrillation, atrial fibrillation, and death.[17] Ventricular tachyarrhythmia including ventricular ectopy, ventricular tachycardia, and ventricular fibrillation are also found in cases of aconite poisoning. Hypotension and bradycardia may also be seen in aconite poisoning. A single dose of aconitine 0.6 mg/kg administered intraperitoneally to rabbits damaged the myelin sheath of the visual pathway, spinal cord, and peripheral nerves. Most of the cardiotoxic and neurotoxic effects of aconite can be explained by these mechanisms, including its effect on calcium imbalance.[18]

Recovery time is dependent on amount of intoxication; mildly intoxicated patients may take 1–2 days to recover, whereas patients with cardiovascular complications may take 7–9 days to recover.[7]

Our patient had consumed *Mahashankha Vati* in which there are eight ingredients and *Vatsanabha (A. ferox* Wall.) is one of them. According to the Ayurvedic formulary of India and *Bhaishajya Ratnavali*, the recommended dose of *Mahashankha Vati* is 125–250 mg and it is indicated in *grahani* (irritable bowel syndrome), *Arsha* (piles), *Mandagni* (anorexia), etc.[19] The constituents of the *Mahashankha Vati* consumed by our patient (500 mg in weight) are shown in [Table 1]. All these ingredients are present in equal amounts in the formulation. Each 250 mg *vati* therefore contains 17.8 mg of aconite.

**Table 1:** Ingredients of *Mahashankha Vati*

*Shudha Parada* (purified mercury)
*Shudha Gandha* (purified sulfur)
*Shudha Vatsanabha* (purified *A. ferox*)
*Tankan Bhasma (sodium biborate)*
*Shankha bhasma (Turbinella rapa* shell)
*Trikatu*
*Shunthi (Zingiber officinale)*
*Maricha (P. nigrum)*
*Pippali (Piper longam)*
*Lavana*
*Saindhaba lavana* (rock salt)
*Vida lavana* (black salt)
*Samudra lavana* (sea salt)
*Souvarchal lavana* (sodium sulfate mixed with sodiumm chloride)
*Oudbhidjja lavana* (ammonium chloride)
*Shudha Hingu* (purified *Ferula asafetida*)

Although the recommended dose of aconite is only 15 mg, our patient had consumed 70 mg of *Vatsanabha (A. ferox* Wall.) by ingesting two tablets of *Mahashankha Vati*. There was a clear temporal relationship, the clinical features were diagnostic of aconite poisoning and there was no other clinical history to explain these features, making a diagnosis of overdose *Vatsanabha (A. ferox* Wall.) very likely. The patient was managed as per the treatment protocol of *Visha chikitsa* (poisoning treatment). *Tankana bhasma* (borax) is mentioned as an antidote of *Vatsanabha* in Ayurveda texts.[8] *Maricha (P. nigrum)* was added as it is a *pramathi* (has a purification effect). *Arjuna (Terminalia arjuna)* has significant positive inotropic properties in experimental models[20] and *Kanakasava* contains atropine. As per Ayurveda, the properties of milk and ghee are opposite to those of poisons. Honey was added as *Yogavahi* (drug efficacy enhancing qualities). As there is no specific antidote for aconite overdosing, we gave symptomatic and supportive treatment,[21] and the patient recovered after 24 hours.

## CONCLUSION

The patient should be aware of the quantity of *Vatsanabha (A. ferox* Wall.) in a formulation and must use such drugs only in the recommended doses and under a physician’s supervision.
